# The application and interpretation of laboratory biomarkers for the evaluation of vitamin B12 status

**DOI:** 10.1177/00045632241292432

**Published:** 2024-10-27

**Authors:** Dominic J Harrington, Emma Stevenson, Agata Sobczyńska-Malefora

**Affiliations:** 1 The Nutristasis Unit, Synnovis, St Thomas’ Hospital, London, UK; 2 School of Biosciences and Medicine, 408263 University of Surrey, Guildford, UK; 3 Department of Clinical Biochemistry, Pathology, 2379 Gloucestershire Royal Hospital, Gloucester, UK; 4 Faculty of Life Sciences and Medicine, 547496 King’s College London, London, UK

**Keywords:** Vitamin B12, active B12, homocysteine, methylmalonic acid, serum B12, NICE

## Abstract

Vitamin B_12_ (cobalamin; B_12_) is an essential micronutrient, but deficiency is common. The prompt diagnosis and treatment of B_12_ deficiency protects against megaloblastic anaemia, neuropathy and neuropsychiatric changes. Biomarkers of B_12_ status include the measurement of serum B_12_ (also known as total B_12_ or serum cobalamin), holotranscobalamin (holoTC or ‘active B12’), methylmalonic acid (MMA) and total plasma homocysteine (Hcy). There is no ‘gold standard’ test for deficiency and the sensitivity and specificity of each biomarker for the evaluation of B_12_ status is affected by analytical and biological factors that may confer a high degree of diagnostic uncertainty. Limited access to technical and clinical expertise can lead to an over-reliance on the serum B_12_ test, which is readily available and highly automated. In some cases, the sequential use of different B_12_ status biomarkers or the calculation of a composite B_12_ status score, derived from a panel of B_12_ biomarkers and adjusted for folate status and age, can be used to detect deficient states that may otherwise be overlooked when using a single biomarker approach. This review summarizes the utility of B_12_-related biomarkers and describes approaches to their application and interpretation.

## Introduction – biochemical relevance and risk factors for deficiency

**Figure 1.**
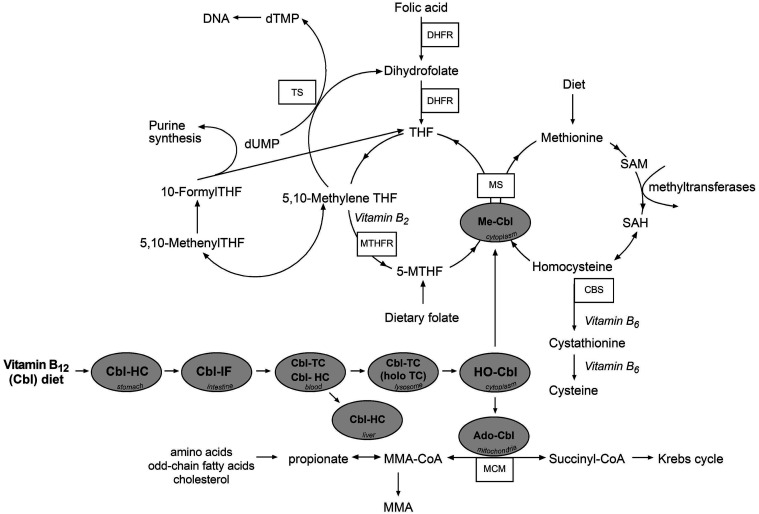
Vitamin B_12_ absorption and intracellular processing via two enzymatic pathways. In the absence of vitamin B_12_, 5-MTHF becomes metabolically trapped in this form producing a *pseudo* folate-deficient state (methyl-trap) and cannot be utilized for regeneration of THF. Cbl, cobalamin; CBS, cystathionine beta-synthase; dTMP, deoxythymidine monophosphate; dUMP, deoxyuridine monophosphate; DHFR, dihydrofolate reductase; HC, haptocorrin; holoTC, holotranscobalamin; HO-Cbl, hydroxocobalamin; IF, intrinsic factor; MS, methionine synthase; Me-Cbl, methylcobalamin; MTHFR, methylene tetrahydrofolate reductase; MMA, methylmalonic acid; MCM, methylmalonyl-CoA mutase; 5-MTHF, 5-methyltetrahydrofolate; SAH, S-Adenosyl homocysteine; SAM, S-Adenosyl methionine; THF, tetrahydrofolate; TS, thymidylate synthase; TC, transcobalamin. Reproduced from reference 3 with permission.

Vitamin B_12_ (cobalamin; B_12_) is an essential micronutrient. In humans, microgram quantities of B_12_ (2–3 µg/d) are required to produce methylcobalamin and adenosylcobalamin, which are used as cofactors in two reactions (Figure 1). One of the reactions takes place in the cytoplasm, where methionine synthase catalyses the transfer of the methyl groups from 5′-methyltetrahydrofolate to homocysteine (Hcy) via the cofactor methylcobalamin to form methionine and tetrahydrofolate. Serum concentrations of Hcy increase in response to a suboptimal supply of methylcobalamin. The other B_12_-dependent reaction takes place in the mitochondria, where the conversion of methylmalonyl-CoA to succinyl-CoA by methylmalonyl-CoA mutase requires the cofactor adenosylcobalamin. A deficiency of adenosylcobalamin leads to an accumulation of methylmalonic acid (MMA), a product of the hydrolysis of excessive methylmalonyl-CoA, which cannot be converted to succinyl-CoA.^1^

In the United Kingdom (UK), the prevalence of B_12_ deficiency is estimated to be up to 20% in some cohorts.^2^ Risk factors for developing deficiency include prolonged omission of B_12_ containing food from the diet, such as meat, fish and dairy, for example, in veganism and vegetarianism; autoimmune gastritis, for example, pernicious anaemia; intestinal diseases; infections or surgical interventions; the increased requirement for B_12_ during pregnancy and in neonatal life; pharmaceutical interactions and nitrous oxide (N_2_O) abuse. In older people, there is a pronounced increase in the prevalence of B_12_ deficiency, mainly caused by gastritis and inadequate dietary intake.^2^

The signs and symptoms of B_12_ deficiency vary considerably from person-to-person. Classical features that may present include anaemia, gastrointestinal and neurological symptoms, and those relating to psychological and psychiatric disturbances.^3^

## Current clinical practice

B_12_ deficiency is most commonly diagnosed and treated in primary care with patients presenting with non-specific complaints, such as tiredness. Clinicians may be guided towards a diagnosis of B_12_ deficiency by the incidental detection of macrocytosis when routine blood tests are performed. However, macrocytosis is a non-specific pathological indicator of advanced B_12_ deficiency and some deficient patients express no haematological abnormality.^4^ Patients should have their B_12_ status evaluated if they have at least one risk factor for deficiency and at least one symptom (Table 1). Clinical judgement should be used when deciding whether to test patients who have no risk factors but at least one sign or symptom.^5^

**Table 1.** An example assessment algorithm for vitamin B_12_ status evaluation. Clinical decision limits for test results are based on those used in the National Institute for Health and Care Excellence (NICE) guidance on vitamin B_12_ deficiency in over 16s: Diagnosis and management (NG239).^5^ Laboratories should evaluate the applicability of clinical decision limits locally.

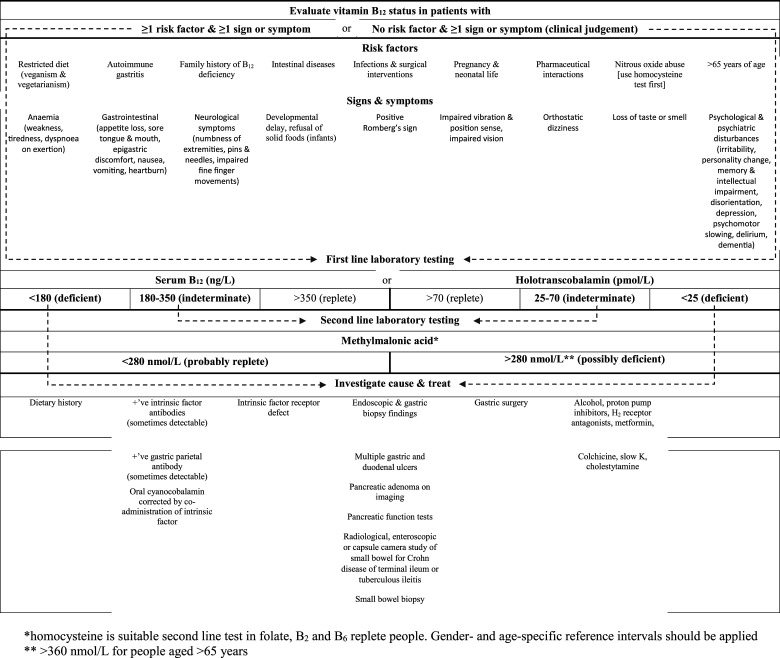

The best characterized biomarkers of B_12_ status are serum B_12_ (also known as total B_12_ or serum cobalamin), holotranscobalamin (also known as holoTC or ‘active B12’), MMA and Hcy.^6^ The utility of B_12_ status biomarkers has not, however, been adequately studied outside of adult populations and there is a need to evaluate their use in children, during each trimester of pregnancy and in some ethnic groups. Audit has shown that a diagnosis of B_12_ deficiency may take several years.^7^ Delays in diagnosis and treatment of B_12_ deficiency have a negative impact on quality of life and increase the likelihood of permanent neurologic damage.^5^ Despite studies consistently demonstrating that no single biomarker of B_12_ status exhibits the performance characteristics necessary to definitively define status in all patients, the majority of diagnostic laboratories rely solely on serum B_12_.

To overcome the performance limitations of individual B_12_ status biomarkers, laboratories should implement first-line testing strategies that maximize diagnostic sensitivity and, where initial test results are indeterminate, second-line testing strategies that maximize diagnostic specificity.^6,8,9^ These strategies are discussed below.

## First-line B_12_ status testing

The high demand in healthcare for B_12_ status investigations limits the selection of first-line biomarkers to serum B_12_ and holoTC, since both are pre-analytically relatively stable and can be performed on highly automated clinical chemistry analysers.

Hcy and MMA are not widely utilized as first-line biomarkers. Hcy is less suitable than serum B_12_ and holoTC because of stringent pre-analytical requirements in which serum/plasma must be separated from whole blood, ideally within 60 min of sample collection, and kept on ice prior to separation to prevent falsely elevated results. Such prompt sample separation is often not available in primary care and Hcy increases by 1 µmol/L in 3 hours in unseparated specimens kept at room temperature.^10^ Alternatively, samples can be collected into tubes containing stabilizing preservatives.^11^ In contrast, MMA samples are pre-analytically stable but high-throughput automated assays for routine laboratories are not yet widely available.

One notable caveat is that neither serum B_12_ nor holoTC have diagnostic utility for the evaluation of B_12_ status in people in whom N_2_O abuse is suspected (refer to Hcy section below).

## Serum B_12_

Since the 1960s, the concentration of B_12_ in serum has been the most commonly used biomarker for the assessment of B_12_ status.^6^ Contemporary serum B_12_ methods are readily available on highly automated clinical chemistry platforms by competitive protein-binding assays using intrinsic factor as the binding protein with chemiluminescence or fluorescence detection systems.^12^

A low serum B_12_ concentration is indicative of deficiency but results within and above the reference interval should be interpreted with an understanding of the limitations of the assay (see also Assay Limitations section below). All serum B_12_ tests detect the total concentration of B_12_, which comprises cobalamin bound to the ß-globulin protein transcobalamin, forming holoTC, and B_12_ bound to the ß-globulin haptocorrin, forming holohaptocorrin (holoHC). Only the B_12_ bound to transcobalamin is transported into cells (holoTC), whereas there is no evidence that B_12_ from holoHC contributes to B_12_ status.^13^ Further, the half-life of holoTC in blood is rapid compared with holoHC, so the majority of B_12_ measured by serum B_12_ assays is holoHC, even though newly absorbed B_12_ mainly binds to transcobalamin. Therefore, serum B_12_ concentrations may not reflect metabolic B_12_ status and the test is considered a late indicator of deficiency when compared with holoTC, Hcy and MMA.

### Result interpretation

Endogenous serum B_12_ concentrations in adults residing in south east London, UK, are ∼182 to ∼692 ng/L (∼134 to ∼511 pmol/L) for Asian and White patients and ∼225 to ∼1091 ng/L (∼166 to ∼805 pmol/L) for Black patients (data generated using the Abbott Architect serum B_12_ assay).^14^ However, variation may be seen across the UK population and with different serum B_12_ assay manufacturers. For example, in the predominantly White patient population of Gloucestershire, UK, endogenous serum B_12_ concentrations in adults are ∼145 to ∼424 ng/L (∼107 to ∼313 pmol/L) when generated using the Beckman DXI platform (unpublished data, author ES). Other published work shows lower reference limits for the Roche and Siemens assays that align well with the authors’ published data using the Abbott Architect assay. A similarly negative bias was also reported when prospectively generating a reference interval using the Beckman DXI platform compared to other assays.^15^

Results from serum B_12_ assays are interpreted against a reference range with the clinical decision point for deficiency commonly set in the region of 200 ng/L (148 pmol/L).^16^ This approach is partly informed by a study in which it was estimated that 90 to 95% of patients with B_12_ deficiency had concentrations <200 ng/L (<148 pmol/L), 5 to 10% had concentrations 200 to 300 ng/L (148 to 221 pmol/L) and less than 1% had concentrations >300 ng/L (>221 pmol/L).^17^ However, results were generated using a radioassay (Quantaphase, Bio-Rad Laboratories, Richmond, CA) and a microbiologic assay using *Lactobacillus leichmannii* rather than assays used in clinical laboratories today. Patients were considered to be B_12_ deficient if they had characteristic features that responded to B_12_ treatment and the authors recognized this as a limitation of their study.^18^

**Figure 2.**
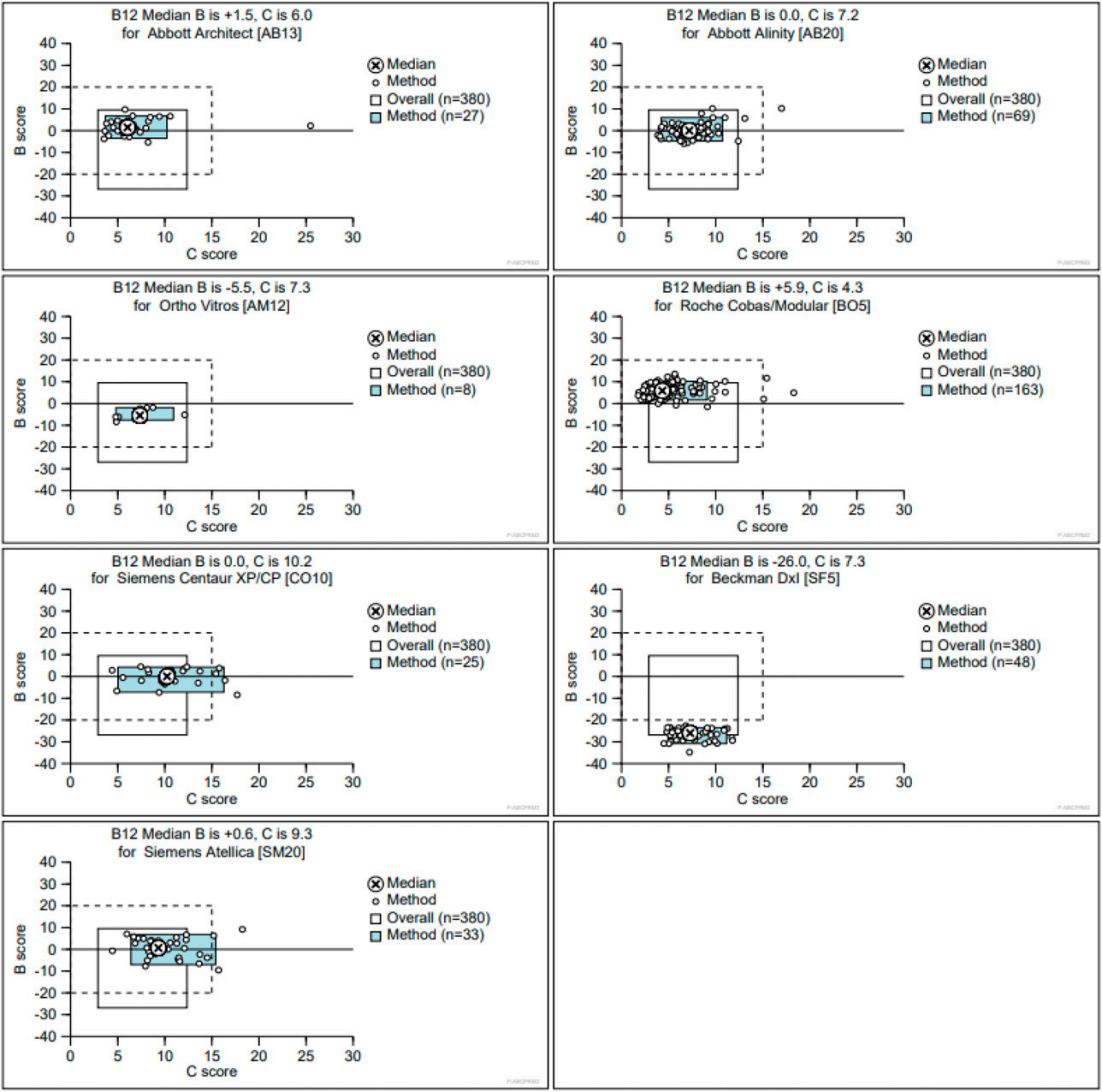
Data from the UK NEQAS Haematinics Scheme showing performance calculated over a rolling window of 6 months (18 External Quality Assurance specimens circulated) by seven analytical methods used for the analysis of vitamin B12 in serum. Methods clockwise from top left [UK NEQAS method abbreviation]: Abbott Architect [AB13]; Abbott Alinity [AB20]; Roche Cobas/Modular [BO5]; Beckman DxI [SF5]; Siemens Atellica [SM20]; Siemens Centaur [CO10]; Ortho Vitros [AM12]. The B score is the average bias of all Specimen % biases [(result – target)/target]×100% during the rolling 6-month window. The C score is the SD of the B score and shows consistency of bias over the same rolling time period. The grey box indicates the 5th to 95th centiles for each method. The unfilled box indicates the overall 5th to 95th centiles irrespective of method. The dotted box indicates limits of acceptable performance defined as ±20% B score and 20% C score. All analyses were performed during October 2023. With permission from Birmingham Quality, University Hospitals Birmingham NHS Foundation Trust.

Results from serum B_12_ assays are interpreted against a reference range or a clinical decision limit for deficiency. Serum B_12_ results are known to be subject to inter-assay variation (Figure 2) and, therefore, manufacturer-dependent validated cut-offs should be used.

Setting a clinical decision limit is a compromise between sensitivity and specificity (Table 2). Consequently, it is good practice to define an indeterminate range between frank deficiency and sufficiency. NICE NG239 suggests an indeterminate range of 180 to 350 ng/L (133 to 258 pmol/L)^5^; however, this will not be appropriate for all serum B12 assays and laboratories should evaluate its applicability locally. Second-line testing should be considered for serum B_12_ results that fall within the indeterminate range.^5,26^

**Table 2.** Summary of studies on the diagnostic performance of serum B_12_, Holotanscobalamin (Active B12), methylmalonic acid, homocysteine and combined indicator of vitamin B_12_ status (4cB12).

Biomarker	Study cohort	Index test of deficiency (decision point)	AUC (95%CI)	Sensitivity (decision point)	Specificity (decision point)	Reference
Serum B12	204 controls & vegetarians	MMA >271 nmol/L	0.836	45% (211 ng/L) 87% (359 ng/L)	98% (211 ng/L) 56% (359 ng/L)	Herrman et al. 2003^19^
	937 individuals with ↑MMA (not treated)	MMA >750 nmol/L	0.85 (0.77 – 0.94)	-	-	Hyas & Nexo 2005^9^
	1279 neurology patients	MMA >397 nmol/L (>47 µg/L)	0.72 (0.65 – 0.78)	-	-	Schrempf et al. 2011^20^
	1359 clinical samples	MMA >300 nmol/L & HoloTC >22 pmol/L	0.632	72% (308 ng/L)	41% (308 ng/L)	Herrmann & Obeid 2013^21^
	5196 clinical samples	MMA >260 nmol/L with ≥50% reduction after B12 replacement	ROC (SE) 0.810 (0.034)	73% (157 ng/L)	74% (157 ng/L)	Bolann et al. 2000^22^
	360 clinical samples	MMA >450 nmol/L	0.63 (0.56 – 0.70) MMA 320 nmol/L 0.70 (0.61 – 0.79) MMA 450 nmol/L 0.73 (0.60 – 0.87) MMA 770 nmol/L	53% (197 ng/L) 64% (244 ng/L)	81% (197 ng/L) 64% (244 ng/L)	Heil et al. 2012^23^
	224 clinical samples serum B12 <300 pmol/L	MMA >376 nmol/L	-	40% (230 ng/L)	98% (230 ng/L)	Hølleland et al. 1999^24^
HoloTC	204 controls & vegetarians	MMA >271 nmol/L	0.879	87% (35 pmol/L)	75% (35 pmol/L)	Herrman et al. 2003^19^
	937 individuals with ↑MMA (not treated)	MMA >750 nmol/L	0.90 (0.83 – 0.97)	-	-	Hyas and Nexo 2005^9^
	1279 neurology patients	MMA >397 nmol/L (>47 µg/L)	0.66 (0.51 – 0.82)	-	-	Schrempf et al. 2011^20^
	1359 clinical samples	MMA >300 nmol/L	0.714	72% (35 pmol/L)	54% (35 pmol/L)	Herrmann & Obeid 2013^21^
	360 clinical samples	MMA >450 nmol/L	0.70 (0.64 – 0.77) (MMA 320 nmol/L) 0.78 (0.69 – 0.87) (MMA 450 nmol/L) 0.92 (0.85 – 0.98) (MMA 770 nmol/L)	83% (32 pmol/L) 64% (21 pmol/L)	60% (32 pmol/L) 88% (21 pmol/L)	Heil et al. 2012^23^
Hcy	5196 clinical samples	MMA >260 nmol/L with ≥50% reduction after B12 replacement	0.768 (SE 0.037)	73% (15.0 mmol/L) 90% (11.3 mmol/L)	68% (15.0 mmol/L) 38% (11.3 mmol/L)	Bolann et al 2000^22^
4cB12	887,871 clinical samples	Subclinical deficiency: 4cB12 ≤−0.5 and >−1.5		Subclinical deficiency: Serum B12	Subclinical deficiency: Serum B12	Campos et al 2020^25^
			Serum B12 (0.9)	86.1% (310 ng/L)	77.7% (310 ng/L)	
				HoloTC	HoloTC	
			HoloTC (0.92)	85.7% (<45 pmol/L)	81.2% (<45 pmol/L)	
				Hcy	Hcy	
			Hcy (0.78)	67.7% (>15 umol/L)	76.7% (>15 umol/L)	
				MMA	MMA	
			MMA (0.91)	81.8% (>245 nmol/L)	83.4% (>245 nmol/L)	
		Possible or probably deficiency: 4cB12 ≤−1.5		Possible or probable deficiency: Serum B12	Possible or probable deficiency: Serum B12	
				94.8% (226 ng/L)	92.3% (226 ng/L)	
				HoloTC	HoloTC	
				93.1% (27 pmol/L)	96% 27 (pmol/L)	
				Hcy	Hcy	
				87.9% (>16.4 µmol/L)	80.9% (>16.4 µmol/L)	
				MMA	MMA	
				94.8% (>466 nmol/L)	96.4% (>466 nmol/L)	

Abbreviations: area under the curve (AUC); reciever operating characteristic (ROC); standard error (SE); methylmalonic acid (MMA); holotranscobalamin (holoTC); homocysteine (Hcy); combined B_12_ status indicator consisting of four biomarkers serum B_12_, holoTC, Hcy and MMA (4cB12).

### Limitations of approach

Age, ethnicity and pregnancy are known to influence serum B_12_ concentrations, yet cohort-matched reference ranges are rarely available to interpret clinical results. Children of all ethnicities have much higher B_12_ concentrations than adults and age-specific reference ranges are warranted.^14,27^ The reasons for much higher B_12_ concentrations in children have not been elucidated.^14^ The application of unified reference ranges in Black patients may lead to an overestimation of B_12_ status because people with Black family backgrounds have significantly higher serum B_12_ concentrations when compared with people with Asian and White family backgrounds.^14^ In pregnancy, serum B_12_ are subject to an ∼50% fall that is related to haemodilution and a decrease in the synthesis of haptocorrin.^28^

### Assay limitations

**Figure 3.**
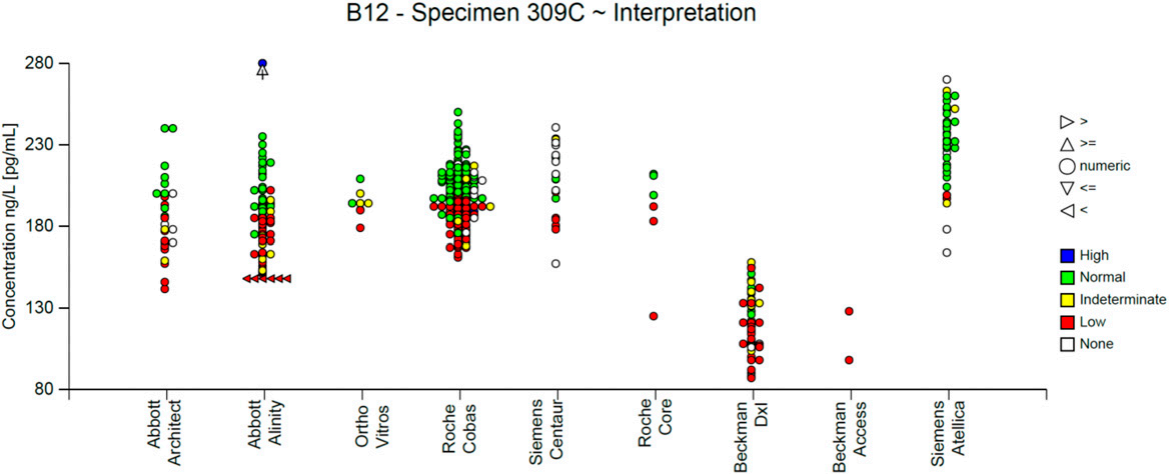
Data from the UK NEQAS Haematinics Scheme showing variation in result interpretation following analysis of distributed aliquots of a single specimen of serum B_12_. All analyses were performed during October 2023 with laboratories applying their local reference range. Interpretation ranges from low B_12_ status to high status. With permission from Birmingham Quality, University Hospitals Birmingham NHS Foundation Trust.

**Figure 4.**
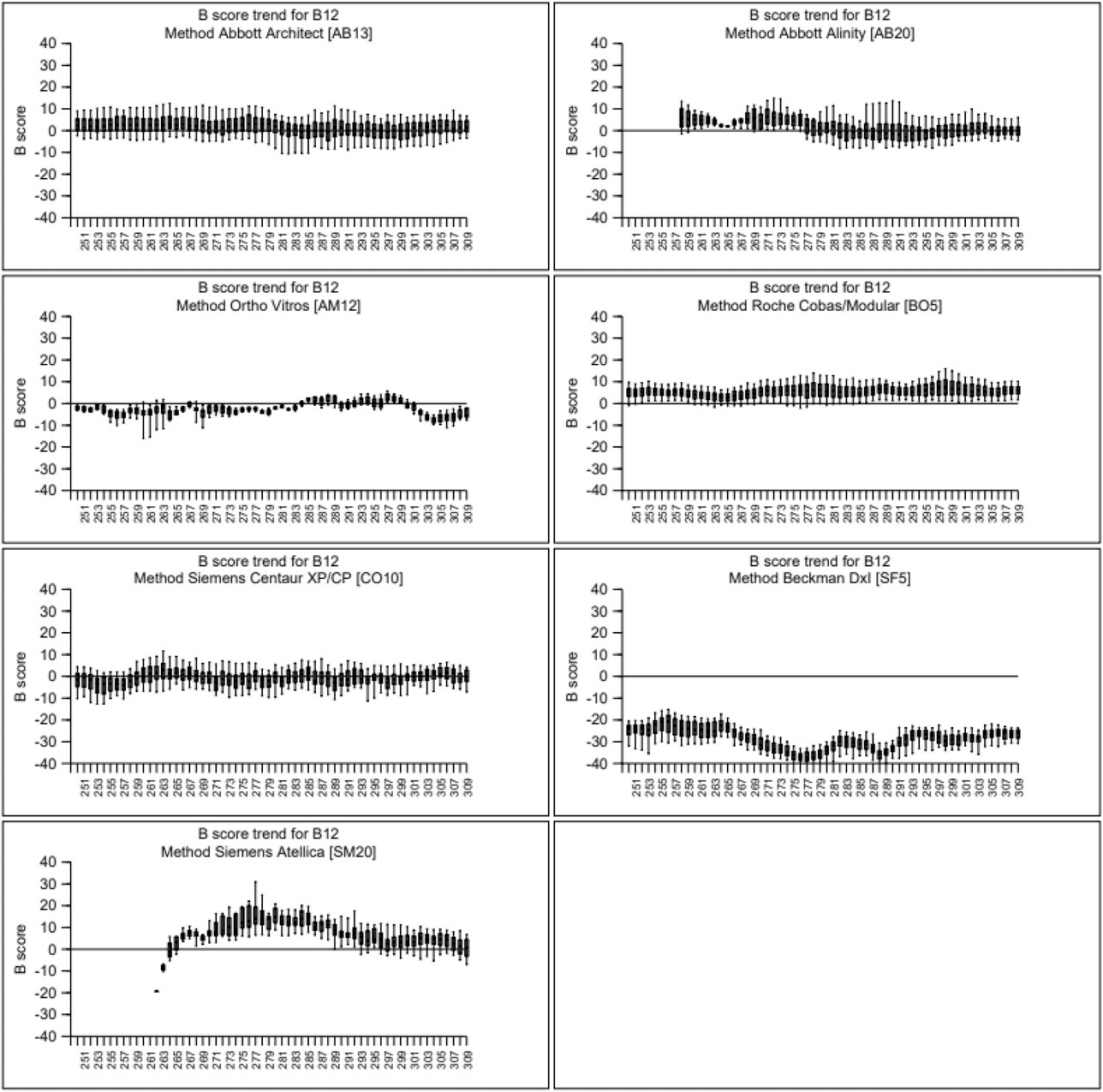
Data from the UK NEQAS Haematinics scheme showing longitudinal bias over a window of 5 years by seven analytical methods used for the analysis of vitamin B12 in serum. Analyses were performed from 2019 to October 2023. With permission from Birmingham Quality, University Hospitals Birmingham NHS Foundation Trust.

The diagnosis of B_12_ deficiency is partly laboratory- and manufacturer-dependent (Figure 3). This unwarranted variation is driven by a lack of harmonization and standardization by manufacturers of serum B_12_ assays.^6^ Serum B_12_ assays are often calibrated independently by manufacturers with traceability to an internally manufactured standard material rather than to an international standard.^6^ Longitudinal variation in the performance of manufacturer-specific assays is revealed through external quality assessment (EQA) performance (Figure 4). Importantly, assay bias and longitudinal variation do not align with the accompanying reference ranges. For example, assays provided by Abbott Diagnostics have higher reference range upper limits, yet it is assays on the Roche platforms that are slightly positively biased when results are compared using EQA. The Beckman systems consistently give lower results than other manufacturers.

False-normal serum B_12_ concentrations have been described in patients with high-titre intrinsic factor antibodies and may also occur because of the presence of heterophile antibody interference.^29^ High haptocorrin concentrations are another cause.^30^

In people with signs and symptoms of B_12_ deficiency who are shown to be B_12_-replete, folate status should be investigated. Coexisting iron deficiency or thalassemia trait may mask macrocytic changes seen on full blood counts.

### Significance of high results

High concentrations of serum B_12_ are frequently seen by clinical laboratories, but seldom investigated or issued with a supporting clinical comment.^31^ High concentrations often reflect B_12_ treatment; however, in some instances, they may be a consequence of underlying liver disease or haematological malignancy.^32^ It has also been demonstrated that immune complexes between serum immunoglobulins and B_12_ binding protein (macro-B12) develop in some individuals that cause assay interference, potentially masking a B_12_ deficient state.^33^ Methods to remove these immune complexes have been described.^34^

## Holotranscobalamin (holoTC, ‘active B_12_’)

The measurement of holoTC in serum provides an estimate of the abundance of B_12_ available for receptor-mediated transportation into cells. HoloTC assays are available on automated clinical chemistry platforms, including those from Abbott (since 2006), Siemens Healthineers (2016), Roche Diagnostics (2017) and Beckman Coulter (2020), at a cost that is higher to perform per test when compared with serum B_12_ assays. If B_12_ is absent from a diet, this will be reflected in the holoTC concentration, which will decrease rapidly in response to the lack of newly absorbed B_12_.^35^

### Result interpretation

A holoTC result of <25 pmol/L indicates deficiency. Receiver operating characteristic curves show HoloTC to be a moderately superior indicator of B_12_ status when compared with serum B_12_ measurement (Table 2). However, in much the same way as serum B_12_, any assigned cut-off point for the interpretation of holoTC assays is a compromise between assay sensitivity and specificity. As an alternative to a single clinical decision point, results in the range of 25 to 70 pmol/L may be considered as indeterminate and second-line testing should be considered to clarify B_12_ status.^5,6,8^ Concentrations >70 pmol/L indicate sufficiency.^5^

Population-specific reference ranges have not been determined. In pregnancy, holoTC is considered the preferred B_12_ status biomarker because, unlike serum B_12_ assays, it is not impacted by the decreased synthesis of haptocorrin.^28,36^

Commercially available assays for holoTC use calibrators traceable to a common primary reference calibrator held by Axis-Shield Diagnostics Ltd, Dundee, Scotland, which confers superior harmonisation when compared with serum B_12_ assays.

## Second-line testing

Second-line testing should be considered when results from first-line testing are indeterminate (see serum B_12_ and holoTC Result Interpretation sections).^5,26^ The best described biomarkers that are considered suitable for second-line testing of B_12_ status are MMA and Hcy.

## Methylmalonic acid

Although no ‘gold standard’ biomarker for the determination of B_12_ status has been identified, MMA is most commonly used as the index test for B_12_ deficiency. Some diagnostic laboratories cite a serum MMA concentration >280 nmol/L as indicative of suboptimal B_12_ status in patients <65 years with normal renal function.^8^ Interpretation is more challenging in the elderly and those with impaired renal function where MMA is likely to be elevated independently of B_12_ status.^37^ A large decrease in MMA concentration after treatment with B_12_ is considered confirmatory of a previously B_12_-deficient state. An MMA concentration >750 nmol/L is accepted as indicative of ‘definite’ B_12_ deficiency.^38^

Unlike Hcy, MMA concentrations are not influenced by folate, vitamin B_6_ or vitamin B_2_ status. Contrasting with the other three biomarkers of B_12_ status, automated MMA immunoassays are not available. An automated LC-MS/MS based assay for MMA analysis is available, however, and capable of processing several hundred samples daily.^12^

In addition to impaired renal function, other B_12_-independent causes of increased serum MMA concentration are states of dehydration, impaired thyroid function, inherited methylmalonic aciduria and small-bowel overgrowth with bacteria producing high amounts of propionic acid, the precursor of MMA.^39,40^ The single nucleotide polymorphism rs291466 in 3-hydroxyisobutyryl-CoA hydrolase (HIBCH) also influences variation in MMA concentration via the valine degradation pathway.^41^

## Homocysteine

The formation of methylcobalamin, to remethylate Hcy to methionine, is dependent on adequate supply of 5′-methyltetrahydrofolate, vitamin B_12_ and vitamin B_2_. The catabolic route of Hcy disposal requires vitamin B_6_. The diagnostic utility of Hcy for the evaluation of B_12_ status therefore rests on the patient being replete for folate, vitamin B_6_ and vitamin B_2_.^42,43^ Assays for the measurement of Hcy are widely available and range from the highly automated immuno- and enzymatic-assays to those that are mass spectrometry-based. Besides rapid separation of plasma/serum from red cells for homocysteine analysis, other factors which may be taken into consideration are post-prandial and orthostatic variations. Samples should ideally be collected after an overnight fast and venepuncture should not be performed after venous stasis or following the subject resting in a supine position.

When interpreting Hcy results, it is recommended that age- and sex-specific reference ranges are applied, although this is seldom done in practice. Children and pregnant women have lower Hcy, while higher concentrations seen in the elderly are likely due to an increased prevalence of intestinal malabsorption, reduced enzymatic activity and reduced kidney function. Adult males have higher Hcy concentrations than females.

Plasma Hcy should be the first-choice test to assess B_12_ status in patients exposed to N_2_O.^3^ The gas is commonly used for sedation and pain relief, but long-term abuse with large doses causes severe B_12_ deficiency, that can present with multiple neurological symptoms and consequences, for example, inability to walk, incontinence, disruption to the reproductive system, depression, demyelinating polyneuropathy and, most significantly, subacute combined degeneration of the spinal cord. Nitrous oxide oxidizes the active cobalt of cobalamin, which eventually leads to dissociation of cobalamin from methionine synthetase (MS) and inactivation of apo-MS. As a result, Hcy cannot be remethylated to methionine. Consequently, Hcy is the first biomarker to respond (increase) in patients who abuse N_2_O. This is followed by a slow decrease in serum B_12_ and holoTC, as well as slight elevations in MMA. Serum B_12_ is often normal at presentation, which may lead to an incorrect differential diagnosis. Although there is no specific marker of N_2_O abuse according to levels of consumption, it has been suggested that MMA may have utility as a marker of clinical gravity.^44^

## Composite biomarker B_12_ status evaluation

A composite score, cB12, which combines the biochemical measurements of serum B_12_, holoTC, MMA and Hcy – while also accounting for low folate status and corrected for age – can be used to evaluate B_12_ status.^45^ The composite score provides an index that relates biomarkers of the individual to the reference combination at the stipulated age. This reference combination has been derived from a large database following mathematical modelling. The formula is expressed as: cB12 = log10 [holoTC × B12/MMA × Hcy] − [3.79/1 (age/230)2.6]. Depending on the cB12 value obtained, B_12_ status is classified as: elevated, adequate, low, possible B_12_ deficiency and probable B_12_ deficiency. Less comprehensive formulas that utilize two or three biomarkers have also been derived (2cB12 or 3cB12) and have been shown to outperform commonly used one-biomarker test approaches.^46^

There have been no large-scale genetic epidemiological studies aimed at describing the basis of variability in cB12 among adults and the composite score is not yet widely used.

## Suggested approaches for the evaluation of B_12_ status

Suggested approaches for the evaluation of B_12_ status are shown in Table 1. Although the diagnosis of B_12_ deficiency is a global challenge, the availability of clinical and technological expertise, as well as resources, vary considerably, and this will influence the approach taken by different healthcare providers.

Blood samples for diagnostic tests should be collected before starting B_12_ replacement. B_12_ replacement treatment should not, however, be delayed while waiting for the test results of people with megaloblastic anaemia or subacute combined degeneration of the spinal cord.

Either serum B_12_ or holoTC assays should be used as the initial test for suspected B_12_ deficiency, unless the test needs to be performed during pregnancy or infancy, or if N_2_O use is the suspected cause of deficiency. HoloTC should be used to assess the B_12_ status of pregnant women and infants, whereas Hcy should be used in N_2_O exposure.

The interpretation of first-line B_12_ tests can be complicated if the person is already receiving supplements since elevated results may reflect recent exposure to B_12_ rather than metabolic B_12_ status.

Deficiency can be confirmed in people with a serum B_12_ concentration below the assay manufacturer-dependent validated cut-off or a holoTC concentration <25 pmol/L.^5^ After exposure to N_2_O, elevated Hcy concentration should be taken as indicative of deficiency; Hcy concentrations >50 µmol/L are often seen in such patients.

MMA measurement should be considered in people who have symptoms or signs of B_12_ deficiency and an indeterminate test result (see Result Interpretation sections).^5^ The cut-offs for serum B_12_ should be applied to adult patients of White and Asian origin. For children, people of Black ethnicity, and in pregnancy, appropriate reference intervals should be used.^14^

B_12_ replacement can commence if an initial B_12_ test result indicates deficiency, or if the initial test is indeterminate in patients with signs or symptoms of B_12_ deficiency and an MMA result is elevated. B_12_ replacement should not be delayed if the total B_12_ result is indeterminate and the patient has a condition or symptom that may deteriorate rapidly and have a major effect on quality of life (e.g. neurological or haematological conditions like ataxia or anaemia), a condition or suspected condition that is an irreversible cause of B_12_ deficiency (e.g. autoimmune gastritis), had surgery that can be an irreversible cause of B_12_ deficiency (e.g. gastrectomy, terminal ileal resection or some types of bariatric surgery) or are pregnant or breastfeeding.^5^

For people with an indeterminate result from first-line testing and no symptoms or signs of B_12_ deficiency, no further testing is required unless signs or symptoms of deficiency develop.

If results from first-line testing for serum B_12_ or holoTC indicate sufficiency, including during pregnancy or breastfeeding, then B_12_ deficiency is unlikely. If, however, symptoms or signs are present 3–6 months later, then the initial test should be repeated.^5^

## Establishing the cause of deficiency

A detailed review of approaches to establishing the cause of a B_12_ deficient state is beyond the scope of this article. A brief summary is provided in Table 1. Establishing the cause of deficiency is important, as in some cases the cause will be found to be reversible and indicate short term treatment is required. In other cases, the cause will be found to be irreversible, leading to obligatory lifelong replacement. B_12_ deficiency is most commonly corrected with oral or intramuscular doses of the vitamin. No toxicity is associated with the treatment.

## Summary

No single laboratory biomarker is suitable for the assessment of B_12_ status in all patients. Using biomarkers in combination leads to a reduction in the number of B_12_-deficient people incorrectly classified as replete and B_12_-replete people incorrectly classified as deficient. In people with a condition or symptom that may progress rapidly, treatment should not be delayed while waiting for results of a laboratory test. Treatment should continue until the cause of B_12_ deficiency has been identified and B_12_ deficiency reversed, or lifelong treatment should continue if the cause is identified as irreversible.
